# A-80426 suppresses CFA-induced inflammatory pain by suppressing TRPV1 activity via NFκB and PI3K pathways in mice

**DOI:** 10.1016/j.clinsp.2023.100213

**Published:** 2023-06-01

**Authors:** Xiaomei Ling, Wei Wang

**Affiliations:** a Department of Anesthesiology, Guangdong Provincial People's Hospital (Guangdong Academy of Medical Sciences), Southern Medical University, Guangzhou, China; b Department of Anesthesiology, Nanfang Hospital, Southern Medical University, Guangzhou, China

**Keywords:** α2-Adrenergic Antagonist, CFA, TRPV1, Pain Behaviors, Inflammation

## Abstract

**Objectives:**

Pain is associated with many circumstances, including inflammatory reactions, which arise from modification of the features of signaling pathways. α2-adrenergic receptor antagonists are widely utilized in narcosis. Here, the authors focused on the narcotic effect of A-80426 (A8) on Complete Freund's Adjuvant (CFA) injections-triggered chronic inflammation pain in WT and TRPV1-/- mice and explored whether its antinociceptive impact was modulated via Transient Receptor Potential Vanilloid 1 (TRPV1).

**Method:**

CFA with or without A8 was co-administered to the mice, which were categorized randomly into four groups: CFA, A8, control, and vehicle. Pain behaviors underwent evaluation through mechanical withdrawal threshold, abdominal withdrawal reflex, and thermal withdrawal latency of WT animals.

**Results:**

Quantitative polymerase chain reaction revealed that inflammation-promoting cytokines (IL-1β, IL-6, and TNF-α) were upregulated in Dorsal Root Ganglion (DRG) and Spinal Cord Dorsal Horn (SCDH) tissues of WT animals. A8 administration reduced the pain behaviors and production of pro-inflammatory cytokines; however, this effect was significantly reduced in TRPV1-/- mice. Further analysis showed that CFA treatment reduced the TRPV1 expression in WT mice and A8 administration increased its expression and activity. The co-administration of SB-705498, a TRPV1 blocker, did not influence the pain behaviors and inflammation cytokines in CFA WT mice; however, SB-705498 the effect of A8 in WT mice. In addition, the TRPV1 block decreased the NFκB and PI3K activation in the Dorsal Root Ganglia (DRG) and Spinal Cord Dorsal Horn (SCDH) tissues of WT mice.

**Conclusions:**

Together, A8 exerted a narcotic impact on CFA-supplemented mice via the TRPV1-modulated NFκB and PI3K pathway.

## Highlights

• A8 treatment inhibits CFA-triggered inflammatory pain.
• A8 treatment exerts a significant inhibitory effect on the production of pain-associated pro-inflammatory cytokines.
• A8 increased the signal transduction of the TRPV1 and CGRP.

## Introduction

α2-adrenoceptor agonists such as detomidine and xylazine are widely accepted as anesthetics by doctors and veterinaries, in order to sedate animals for a comparatively long period [1]. Dexmedetomidine (DEX) is employed for the narcosis and extradural management of intractable pain; it binds to α2-receptors with stronger affinity (8-fold higher) than clonidine. Previous research has shown that the narcotic effect of DEX is greater than that of clonidine, and DEX's antinociceptive effect is observed after intraspinal treatment. It has been previously shown that co-treatment of DEX and gabapentin can synergistically counteract inflammation pain triggered via intra-articular injection of Complete Freund's Adjuvant (CFA); however, a reduced dosage of DEX or gabapentin alone had little impact on Paw Withdrawal Latencies (PWLs) of the hindpaw on the same side as CFA-injected joint [2]. Intrathecal DEX injection leads to short-term narcosis in a dose-dependent manner, and continuous everyday treatment of their combination displays synergistic narcosis and noticeably represses neuron and astrocyte stimulation, triggered via CFA injection [3]. Although the effect of α2-adrenoceptor agonists on chronic pain induced by CFA has been previously reported, the role of α2-adrenoceptor antagonists during this period is still unknown. A-80426 (A8), also known as (N-[2-(Benzofuran-6-yl)ethyl]-N-[I-5-methoxy-1,2,3,4-tetrahydronaphthalen-1-ylmethyl]-N-methylamine), exhibits potent α2-adrenoceptor antagonist activity with equivalent serotonin 5-HT uptake repressive function [4].

Some channels, signaling agents, and receptors in microglia and neuronal cells mediate pain transmission. Transient Receptor Potential Vanilloid 1 (TRPV1) of ion channel exerts a crucial impact on not only nociceptive [5], but also on neuropathic algesia [6]. TRPV1 expression is found in peripheral Dorsal Root Ganglion (DRG), the brain, and central SCDH (Spinal Cord Dorsal Horn). The PI3K/AKT/mTOR (mTORC1) axis contributes to cellular immunity [7]. Furthermore, TRPV1 stimulation upregulates the expression levels of PI3K, CREB, Nav1.7, AKT, NF-κB, and Nav1.8. Such upregulation is repressed in TRPV1-/- mice [8]. Thus, the authors assessed the effect of A8 on the CFA-triggered inflammatory pain in WT and TRPV1-/- mice. The present research offers innovative insights into the association between α2-adrenoceptor agonist A8, inflammatory pain, TRPV1, and NFκB and PI3K pathways. Surprisingly, the present data showed that administration of A8 also exerted an antinociceptive function on CFA-induced inflammation pain. The further study explained that TRPV1 is responsible for the anti-inflammatory and antinociceptive of A8. Therefore, the authors hypothesized that A8’s antinociceptive role is TRPV1 dependent, rather than α2-adrenoceptor.

## Materials and methods

### Experimental animals

Mice were managed in accordance with the National Institute of Health Guide for the Care and Use of Laboratory Animals. Protocol was approved by the ethics committee of the Experimental Animals Center of Southern Medical University (n° NFYY-2021-0214). All the animal experiments were followed the ARRIVE guidelines and conducted in the Animal Experimental Center of Nanfang Hospital.

C57/B6 mice and TRPV1-/- mice, eight to twelve weeks of age and weighing nearly 25g, were bought from Vitalriver Animal Center and kept within Plexiglas cages at 25±2°C with a humidity of 60%±5%. Standard mouse chow and water were freely available. Mice were starved but were freely provided with water 2 hours prior to experiment. Four percent chloral hydrate (200 mg per kg body) was administered for mouse anesthesia via intraperitoneal injection. Mouse went into a deep sleep is the major parameter to ensure the mice were fully anesthetized. After experiments, mice were humanely sacrificed by spinal dislocation after anesthesia according to animal ethics guidelines.

### Inflammatory pain model

Each group consisted of at least eight mice according to previous research [8]. Mice were then divided into 4 categories: (1) Control group: normal saline injection, (2) CFA group: CFA injection (to trigger inflammatory pain), (3) A8 group: CFA injection and A8 manipulation, and (4) Vehicle group: CFA injection and vehicle manipulation in order to investigate the contribution of TRPV1 to inflammatory pain. (5) SB group: CFA injection and SB-705498 administration in order to investigate the contribution of TRPV1 activation to inflammatory pain. (6) A8+SB group: CFA injection and SB-705498 administration in order to investigate the contribution of TRPV1 activation to A8-alleviated inflammatory pain. Every procedure was conducted in the laboratory during daytime. Two A8 interventions were conducted at 24h and 48h after CFA treatment.

Behavior exams and the Hargreaves test were utilized in order to evaluate thermal and mechanical hyperalgesia at baseline, immediately after CFA injection and 24h and 48h after CFA treatment. Pain-repressing impacts of A8 and TRPV1 knockout were compared 2d after CFA treatment and A8 administration. One percent isoflurane was applied to murine narcosis. Twenty microliters of CFA (provided by Sigma, St. Louis; 0.5 mg/mL heat-treated *M. tuberculosis*) or saline (buffered by 20 mM HEPES; Ph 7.4) was injected into plantar surface of the hind paws in order to trigger intraplantar inflammatory reactions.

### Treatment with α2-adrenergic antagonist, A-80426 (A8), and TRPV1 blocker, SB-506498 (SB)

Eight C57BL/6 male mice aged from eight to twelve weeks were utilized for this experiment. Twenty-four hours after the triggering of inflammatory reactions, 100 µL of saline containing A8 (and/or SB) was injected intraperitoneally (i.p.) daily at a dose at 10 mg/kg. Ten percent DMSO was utilized in order to dissolve A8 (50 μg/100 μL, Sigma), or SB (85 μg/100 μL, Alomone Labs). The vehicle control group was injected with 10 µL of 10% DMSO.

### Abdominal withdrawal reflex (AWR), mechanical withdrawal threshold (MWT), and thermal withdrawal latency (TWL) measurements

AWR was evaluated with the help of Colorectal Distending (CRD) with four grades of triggers: 80, 60, 40, and 20 mmHg. The activation time of every CRD was the 20s, and a 5-min interval existed between two triggers. Three examinations were conducted independently, and the average was evaluated.

MWT was determined by stimulating the center of the posterior paw for less than 4s with a von Frey filament. Raising or biting the stimulated paw was defined as a positive response. The lowest load was 2g and the highest load was 15g. Every trigger was repeated five times, and there was a 30s gap between successive examinations.

A thermal stimulator was used to determine TWL, which refers to the interval between the beginning of the thermal radiation and the withdrawal behavior. The stimulation was conducted with a preset intensity, which was reduced after 20s to prevent thermal damage to the skin.

### Tissue sampling

Mice were executed via CO_2_ euthanasia method in order to reduce pain. L3‒L5 Dorsal Root Ganglia (DRG) and Spinal Cord Dorsal Horn (SCDH) were obtained 48h after CFA treatment and were cut immediately to isolate proteins. Lysis buffer containing 50 mM Tris-HCl (pH 7.4), 1% NP-40, 50 mM NaF, 0.02% NaN_3_, 250 mM NaCl, 5 mM EDTA, 1 mM Na3VO4, and 1 × protease inhibitor cocktail (AMRESCO) was used in order to isolate proteins in the homogenized DRG and SCDH tissue.

### Western blotting (WB)

Isolated proteins (30 µg from every specimen as evaluated via BCA protein assay) were subjected to electrophoresis (8%‒12 % SDS-Tris glycine gel) and were transferred to the PVDF membranes. Membranes were blocked with the help of 5% skimmed milk within TBS-T buffer (10 mM Tris [pH 7.5], 100 mM NaCl, 0.1% Tween 20) and incubated with primary antibodies (GAPDH antibody [1:5000, ab8245, Abcam], IL-1beta antibody [1:5000, ab9722, Abcam], IL-6 antibody [1:2500, ab6672, Abcam], TNF-alpha antibody [1:2500, ab6671, Abcam], TRPV1 antibody [1:1000, ab10296, Abcam], phosphor-AKT antibody [1:1000, ab66138, Abcam], AKT antibody [1:5000, ab8805, Abcam], PI3K antibody [1:2000, ab86714, Abcam], phosphor-p65 antibody [1:1000, ab86299, Abcam], and p65 antibody [1:5000, ab16502, Abcam]) at room temperature. The membranes were then incubated with secondary antibodies, which were anti-rabbit antibodies (1:5000) conjugated with peroxidase. Bands were observed via an enhanced chemiluminescent substrate kit (provided by PIERCE) with the help of LAS-3000 Fujiflm (Fuji Photo Film Co. Ltd). Band intensity was quantified using ImageJ software (Bethesda, MD, USA).

### Isolation of RNA and real-time polymerase chain reaction (PCR)

Trizol agent (Invitrogen) was utilized in order to isolate RNA from tissues according to the manufacturer's protocol and subsequently quantified using NanoDrop™ 2000 machine (Thermo Fisher Scientific, Inc.). The synthesis of cDNA was performed using the PrimerScript™ RT Reagent kit (Takara Bio Inc.) and qPCR was conducted by the FastStart Universal SYBR Green Master kit (Roche Diagnostics) with the help of LightCycler 480 real-time PCR system (provided by Roche, Germany). Quantitative PCRs (qPCRs) were carried out with a 20 µL reaction volume containing SYBR Green PCR Master Mix. The thermal cycling conditions for PCR were: Initial denaturation at 95°C for 15 min, followed by 35 cycles of denaturing at 95°C for 15 sec, annealing at 55°C for 30 sec and elongation at 72°C for 30 sec. GAPDH was recognized as the internal control. mRNA levels were quantified using 2^−ΔΔct^ approach and normalized against GARDH levels.

### ELISA

The concentration of Calcitonin Gene-Related Peptide (CGRP) was assessed using an ELISA kit (provided by R&D Systems, Minneapolis, USA). Absorbance was observed at 543 nm with the help of a microplate reader (provided by BMG Labtech, Offenburg, Germany).

### Immunohistochemistry (IHC) analysis

Paw tissues were excised, fixed in 10% formalin buffer at 4°C overnight, then dehydrated, cleared, and embedded in paraffin. Tissues were sectioned (4 mm), flattened, and adhered to glass slides. After dewaxing and dehydration, antigen recovery was performed by incubating the sections in Ph 6.0 citric acid buffer for 10 min in 98°C deionized water. After cooling at room temperature for 20 min, the sections were immersed in 3% hydrogen peroxide for 15 min at room temperature to abolish peroxidase activities. The sections were incubated with primary antibodies (TRPV1 antibody, 1:1000) in PBS for 1h at room temperature. Then, the sections were washed with PBS and incubated for 30 min with a secondary antibody at room temperature. After washing with PBS, the sections were stained with 3,3′-Diaminobenzidine peroxide (DAB) chromophore, counterstained with hematoxylin, and mounted on microscope slides for analysis. Brown staining in the cytoplasm indicated positive staining for TRPV1. The mean percentage of positively stained cells was determined by counting 1,000 stained cells in 10 different fields under 200 × magnification using a light microscope.

### Statistical analysis

The results were displayed in the form of mean ± SD; p-value < 0.05 was regarded as statistically significant. A two-way ANOVA test was used to evaluate the difference between multiple groups, followed by post hoc Tukey's test. SPSS for Windows (Version 21.0 SPSS, Chicago, America) was used for statistical analysis.

## Results

### Effects of A8 treatment on AWR, MWT, and TWL of CFA-injected WT mice

**Fig. 1:**
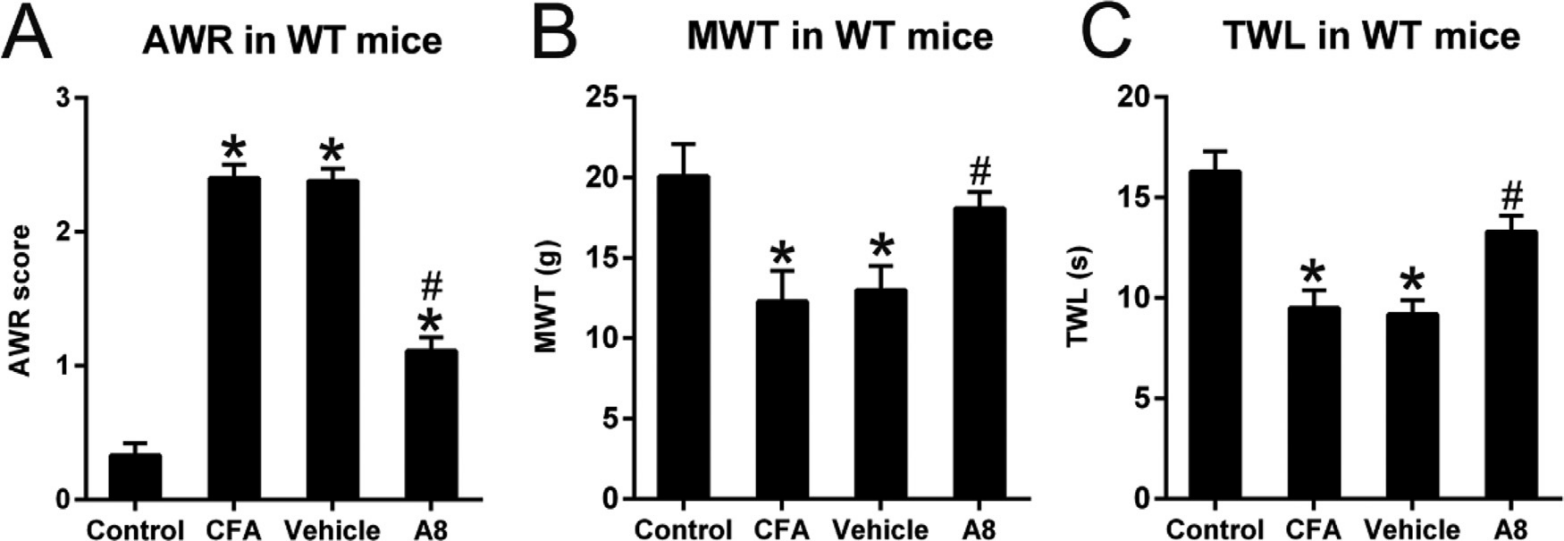
**Effects of A8 administration in the CFA-induced WT mouse**. (A) Post-treatment AWR figures in WT mice. (B) MWT subsequent to DEX treatment in WT mice. (C, F) TWL value following DEX treatment in WT mice. The results of the three separate tests were presented as mean ± SD. *n* = 8 mice in each group. ^#^*p* < 0.05, compared to the CFA group; *p < 0.05, compared to control group.

After treatment with CFA in WT mice, the AWR scores (2.40 ± 0.10) of CFA-treated animals significantly increased relative to those of normal controls (0.33 ± 0.09, *p* = 0.0129) (Fig. 1A). In contrast, TWL (9.5 ± 0.9) and MWT (12.3 ± 1.9) values of treated animals were significantly decreased compared to those of untreated animals (*p* = 0.0232 and 0.0332, respectively) (Fig. 1B, C). Further, A8 administration induced lower AWR scores (1.11 ± 0.10) in the A8 group relative to those in the CFA and vehicle groups (2.38 ± 0.09, *p* = 0.0375) (Fig. 1A). In addition, MWT (18.1 ± 1.0) and TWL (13.3 ± 0.8) values were elevated in the A8 group compared to those in the CFA and vehicle groups (*p* = 0.0431 and 0.0395, respectively) (Fig. 1B, C). These data suggest that A8 administration influenced the pain behaviors of CFA-induced mice.

### Effects of A8 treatment on inflammatory responses of CFA-injected WT mice

**Fig. 2:**
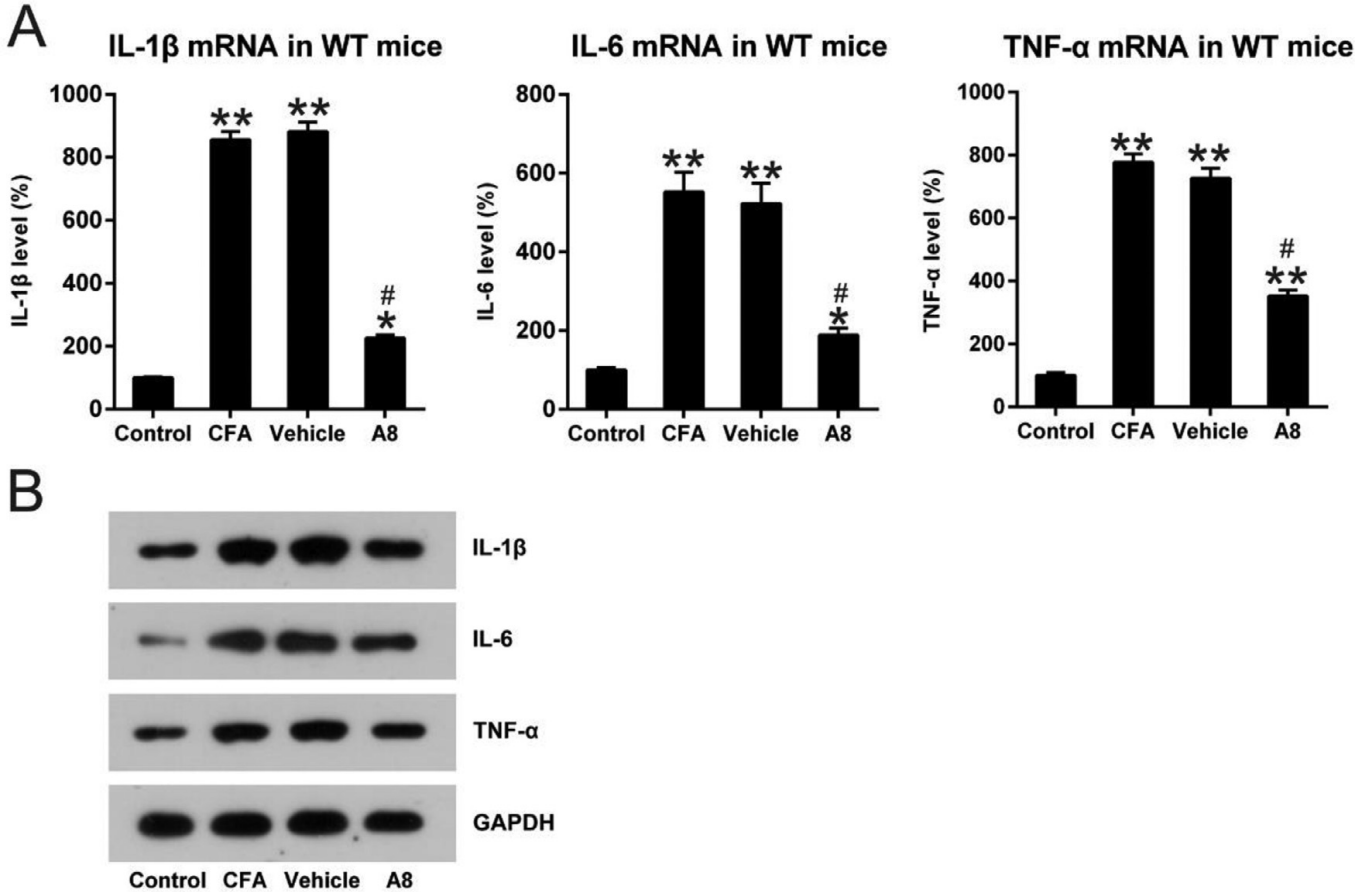
**Impact of A8 on inflammatory cytokine production in the CFA-induced WT mouse**. Forty-eight hours after injection of CFA, tissue was collected from the WT mice, and then, IL-1β, IL-6, and TNF-α mRNA levels were determined by (A) qPCR and (B) WB. The results of three independent experiments were presented as mean ± SD. *n* = 8 mice in each group. ^#^*p* < 0.05, compared to the CFA group; *p < 0.05, **p < 0.01, compared to the control group.

To evaluate the influence of A8 on the production of pro-inflammatory cytokines, mice were treated with various doses of A8 or vehicle, followed by induction of an inflammatory response by CFA. qPCR was performed to assess the mRNA levels of pro-inflammatory cytokines, such as IL-1β, IL-6, and TNF-α, under these conditions. As shown in Fig. 2A, compared to the control groups, expression levels of IL-1β (856.3 ± 26.5), IL-6 (552.7 ± 50.5), and TNF-α (777.0 ± 52.2) significantly increased in the CFA group (*p* = 0.0081, 0.0036, and 0.0087, respectively). However, treatment with A8 resulted in an apparent downregulation of IL-1β (226.4 ± 19.2), IL-6 (189.7 ± 23.7), and TNF-α (365.9 ± 18.5) expression. The data from WB is consistent with that from qPCR (*p* = 0.0221, 0.0386, 0.0443, respectively) (Fig. 2B). These results demonstrated that inflammation induced by CFA was ameliorated by the administration of A8.

### Expression and activity of TRPV1 in the CFA-induced and A8-treated mice

**Fig. 3:**
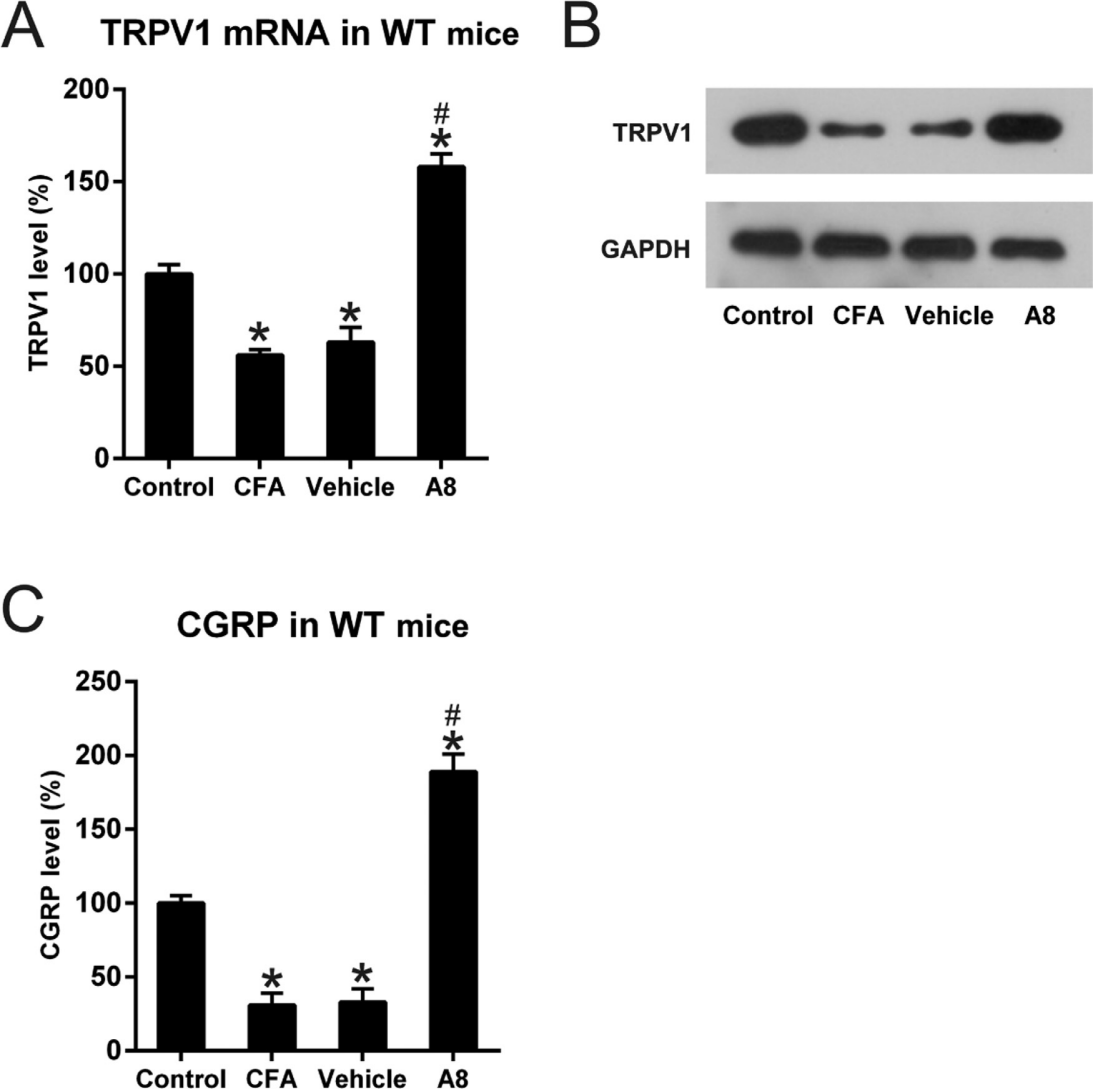
**Effects of CFA and A8 treatment on expression and activity of TRPV1 channel in mice**. (A) qPCR and (B) WB analysis determined mRNA levels of TRPV1 of mice in vehicle, CFA, A8, and control groups. (C) ELISA was employed to assess the levels of CGRP in DRG and SCDH tissues of mice in vehicle, CFA, A8, and control groups. The results of three separate experiments were presented as mean ± SD. *n* = 8 mice in each group. ^#^*p* < 0.05, compared to the CFA group; *p < 0.05, compared to the control group.

Next, the authors used qPCR and WB to investigate whether TRPV1 participated in CFA-mediated induction of inflammatory pain and to measure the modulating capability of A8 treatment on this mouse model. In the first step, tissue lysates from each group were examined for the expression of TRPV1. As shown in Fig. 3A and B, TRPV1 mRNA and protein level was significantly downregulated following CFA treatment, which was later increased by A8 administration (*p* = 0.0238). The authors also examined the activity of TRPV1 channel, which was able to be determined by the measuring release of CGRP. As shown in Fig. 3C, ELISA data confirmed that the CGRP level was decreased after CFA treatment (*p* = 0.0345), whereas A8 treatment significantly promoted the activity of the TRPV1 channel (*p* = 0.0233).

### Effects of TRPV1 knockdown on A8-ameliorated pain behavior and inflammatory response of CFA-injected mice

**Fig. 4:**
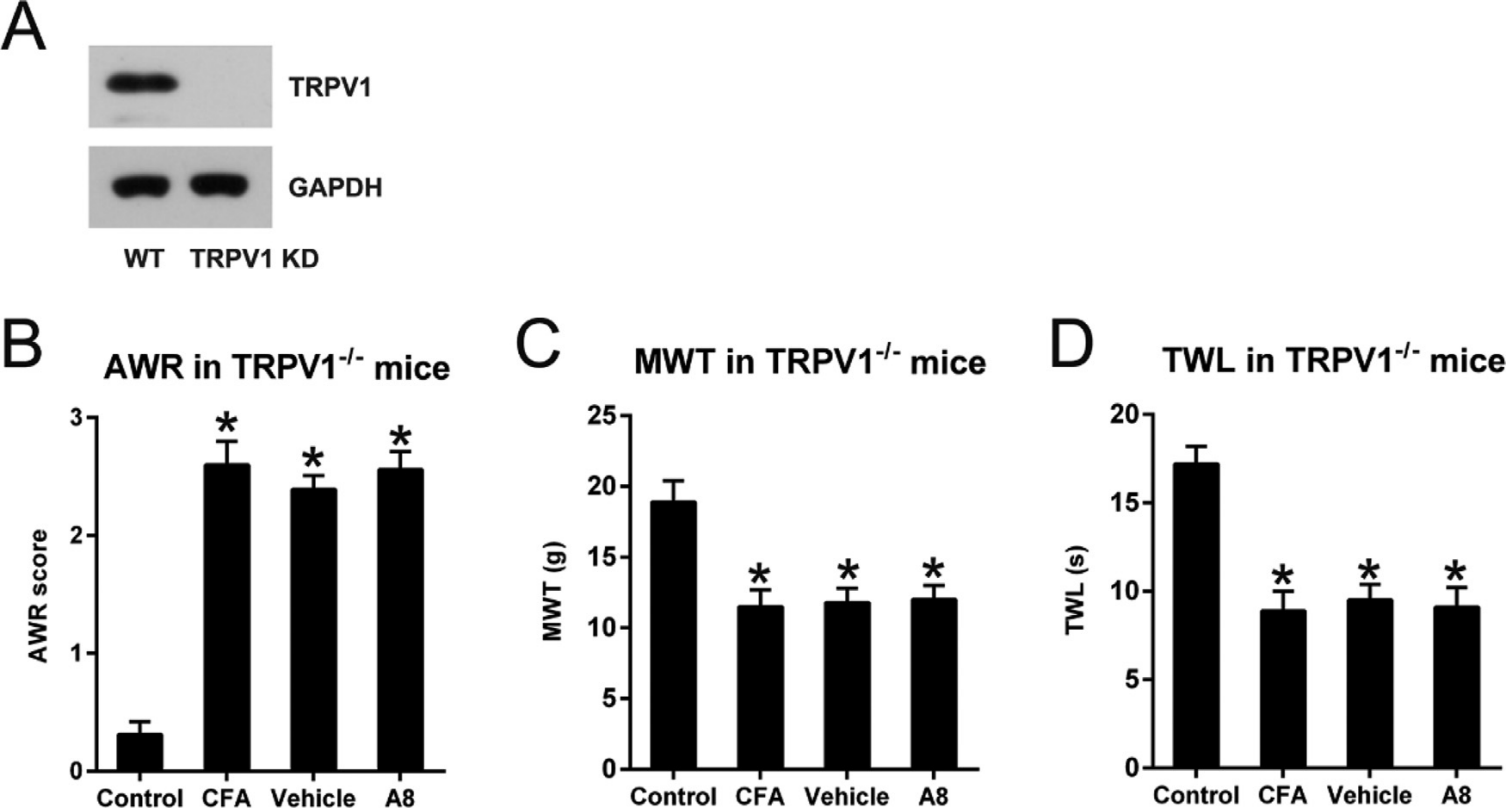
**Effects of A8 administration in the CFA-induced TRPV1-/- mouse**. (A) WB determined the protein expression of TRPV1 in WT and TRPV1-/- mice. (B) Post-treatment AWR figures in TRPV1-/- mice. (C) MWT subsequent to DEX treatment in TRPV1-/- mice. (D) TWL value following DEX treatment in TRPV1-/- mice. The results of the three separate tests were presented as mean ± SD. n=8 mice in each group. *p < 0.05, compared to control group.

To further confirm the importance of the TRPV1 channel in CFA-treated inflammatory pain mice, the authors also utilized the TRPV1-/- mice to create the inflammatory pain model. The knockdown of TRPV1 was confirmed as assessed by WB examination (Fig. 4A). These TRPV1-/- mice were subjected to the same CFA and A8 treatment and grouping as the WT mice. Behavioral tests were also performed. The authors found that TRPV1 knockout did not cause a significant difference in AWR (26.1 ± 0.22), TWL (8.9 ± 1.2), and MWT (11.5 ± 1.6) in the CFA group compared to WT mice (*p* = 0.0103, 0.0422, and 0.0287, respectively). A8 treatment did not result in significant alteration of AWR (2.56 ± 0.18) scores of TRPV1-/- mice compared to CFA and vehicle group (2.39 ± 0.12, p > 0.05) (Fig. 4B). Notably, TWL (9.1 ± 1.5) and MWT (12.3 ± 2.0) values of TRPV1-/- mice were not significantly altered between both CFA group and A8 treatment group (p > 0.05) (Fig. 4C, D).

**Fig. 5:**
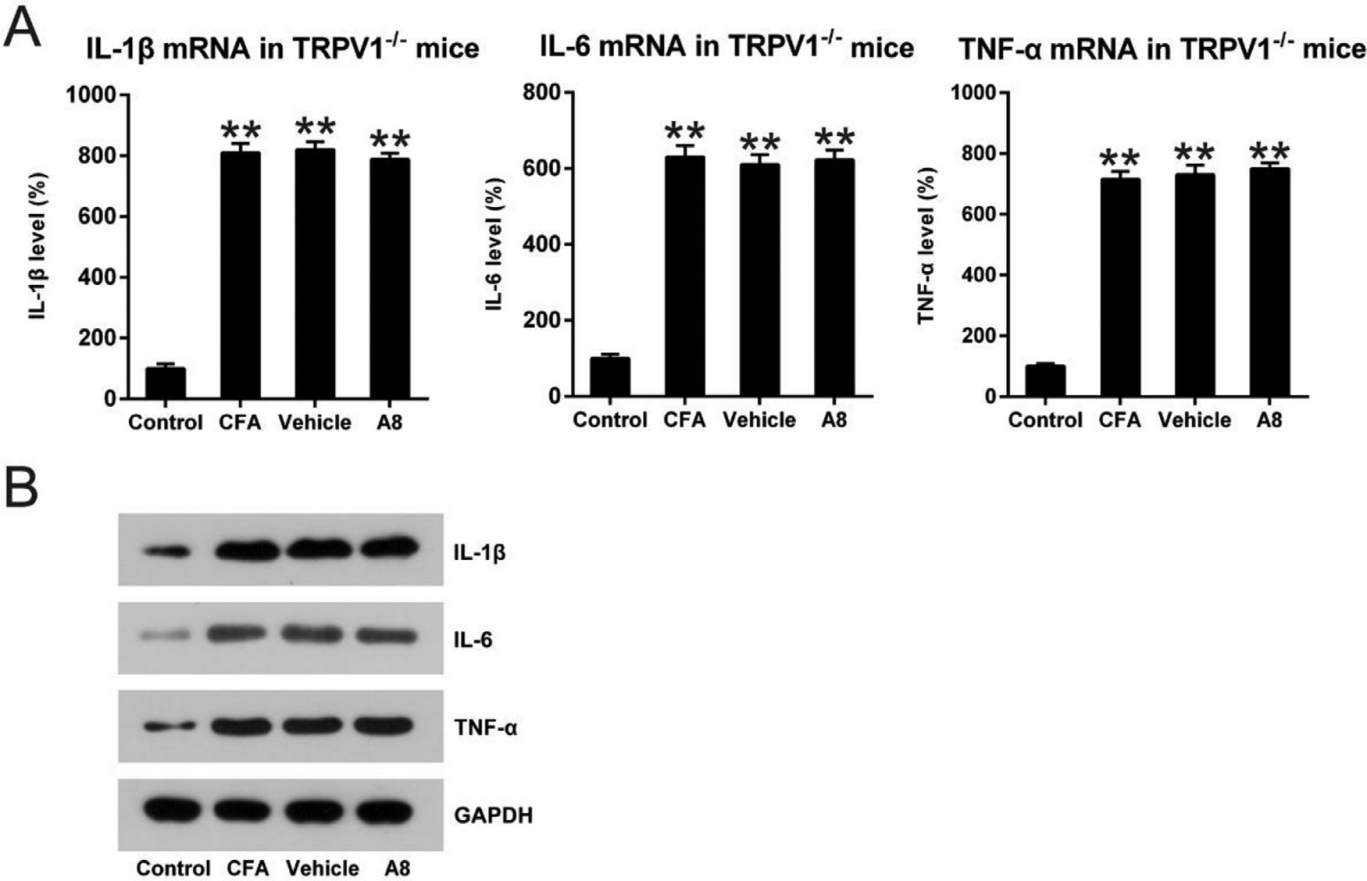
**Impact of A8 on inflammatory cytokine production in the CFA-induced TRPV1-/- mouse**. Forty-eight hours after injection of CFA, tissue was collected from the TRPV1-/- mice, and then, IL-1β, IL-6, and TNF-α mRNA levels were determined by (A) qPCR and (B) WB. The results of three independent experiments were presented as mean ± SD. *n* = 8 mice in each group. **p < 0.01, compared to the control group.

To further confirm the influence of TRPV1 in the inflammation of CFA-injected mice, qPCR and WB were performed to assess mRNA levels of IL-1β, IL-6, and TNF-α. As shown in Fig. 5A, B, expression levels of these cytokines were significantly improved for both WT and TRPV1-/- mice after CFA administration (p=0.0085, 0.0093, and 0.0087, respectively). Meanwhile, in the TRPV1-/- mice, treatment with A8 did not result in an apparent downregulation of IL-1β, IL-6, and TNF-α (p>0.05). The results demonstrate that acute inflammation downregulated by A8 is dependent on TRPV1 expression.

### TRPV1 inhibition suppressed the effect of A8 on CFA-treated mice

**Fig. 6:**
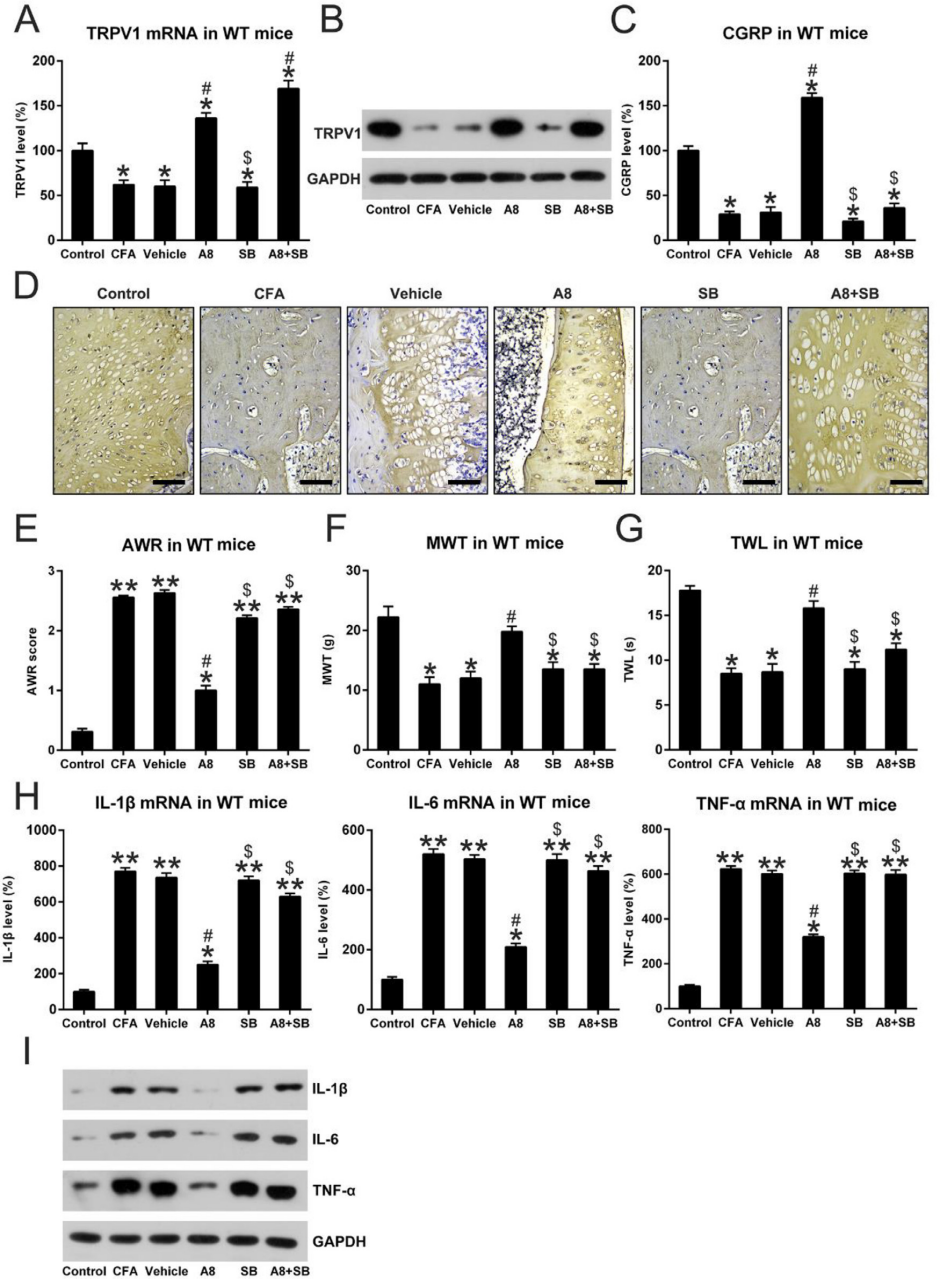
**Effects of SB treatment on expression and activity of TRPV1 channel, pain behavior, and inflammation in mice**. (A) qPCR and (B) WB, (C) IHC was done to assess mRNA levels of TRPV1 of mice in vehicle, CFA, A8, SB, A8+SB, and control groups. Scale bar for IHC, 200 μm. (D) ELISA was employed to assess the levels of CGRP in DRG and SCDH tissue of mice in vehicle, CFA, A8, SB, A8+SB, and control groups. (E) Post-treatment AWR data of mice in vehicle, CFA, A8, SB, A8+SB, and control groups. (F) MWT values of mice in vehicle, CFA, A8, SB, A8+SB, and control groups. (G) TWL values following DEX treatment in WT and TRPV1-/- mice. (H, I) Forty-eight hours after administration of CFA, tissues were collected from the animals, and then, IL-6, IL-1β, and TNF-α mRNA levels was determined by qPCR and WB. The results of three separate experiments were presented as mean ± SD. *n* = 8 mice in each group. ^#^*p* < 0.05, compared to the CFA group; *p < 0.05, **p < 0.01, compared to the control group; ^$^*p* < 0.05, compared to the A8 group.

To further probe the involvement of the TRPV1 in A8-treated mice, the authors also co-treated the mice with SB-705498 (SB), a blocker of TRPV1 channels, to inhibit TRPV1 activity which was promoted by A8 administration [9]. These CFA mice underwent the same A8 treatment but were also co-treated with SB. At first, the levels of TRPV1 and CGRP release were determined after A8 and/or SB administration. The qPCR and WB data showed that TRPV1 expression was not altered by SB treatment; however, SB treatment clearly led to the downregulation of CGRP level (Fig. 6A, B). To further confirm the expression of TRPV1 in each group, the protein expressions of TRPV1 were determined using IHC. As shown in Fig. 6D, the CFA-induced inflammation group showed significantly decreased expression of TRPV1 compared to the control group (p=0.0256). As expected, treatment with A8 significantly upregulated TRPV1 expression when compared with the vehicle group (*p* = 0.0356), while co-treatment with SB did not obviously affect the TRPV1 expression (p > 0.05) indicating that SB treatment only repressed the activity of TRPV1 without affecting TRPV1 expression.

To reveal the effect of TRPV1 blockage on pain behavior in CFA-induced mice, they were subjected to the same behavioral tests. The authors found that TRPV1 inhibition led to higher AWR scores of the SB model (2.21 ± 0.12) and A8+SB groups (2.36 ± 0.50) compared to those of A8 mice (1.02 ± 0.08, *p* = 0.0320 and 0.0258, respectively) (Fig. 6E). Moreover, TWL (9.0 ± 0.8 and 11.2 ± 0.9) and MWT (13.5 ± 1.5 and 13.8 ± 1.6) values of mice in SB model and A8+SB groups were also significantly decreased compared to A8 group (all *p* < 0.05) (Fig. 6F, G). These data suggested that SB administration influenced the pain behaviors of CFA-induced mice, at least partially, in a TRPV1 channel-dependent manner.

To detect the influence of TRPV1 blockage on CFA-administered mice, the authors examined the generation of pro-inflammatory cytokines within each group. qPCR and protein were performed to assess mRNA and protein levels of IL-1β, IL-6, and TNF-α. Expression levels of these cytokines were significantly upregulated in both SB and A8+SB groups (*p* < 0.05). Meanwhile, in the SB-administered mice, treatment with A8 did not result in an apparent downregulation of IL-1β, IL-6, and TNF-α (Fig. 6H, I). The results demonstrated that acute inflammation downregulated by A8 was dependent on TRPV1 activation.

### A8 and SB administration regulated the NFκB and PI3K pathway in CFA treated mice

**Fig. 7:**
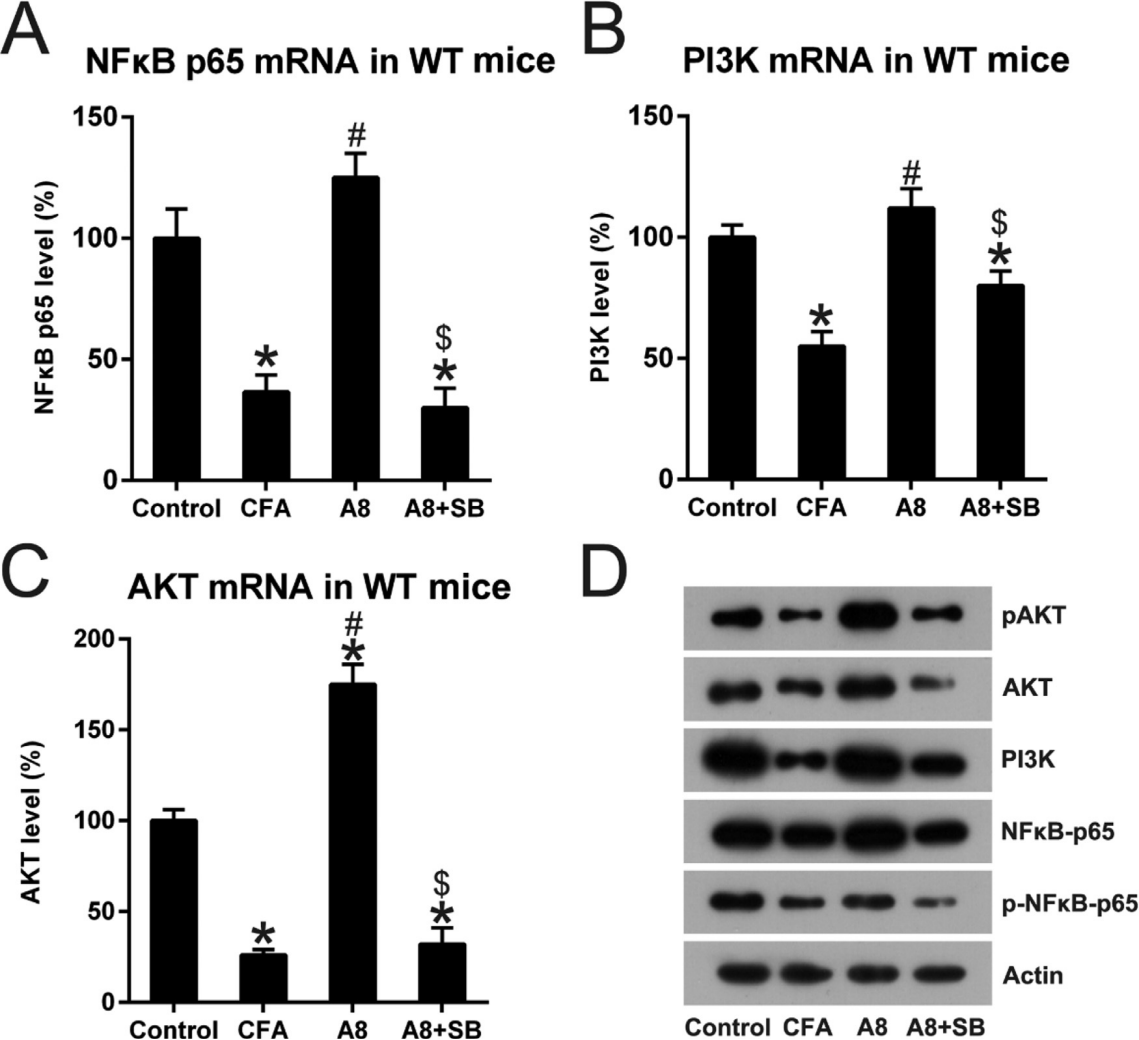
**Impact of TRPV1 blockage on NFκB and PI3K activation**. Forty-eight hours after injection of CFA, tissue was collected from the animals. (A, B, C) qPCR was employed to examine mRNA levels of NFκB, PI3K, and AKT in the DRG and SCDH tissue of mice in CFA, A8, A8+SB, and control groups. (D) WB was employed to determine the protein levels of NFκB, PI3K, and AKT, and phosphorylation of NFκB and PI3K, in the DRG and SCDH tissues of mice in CFA, A8, A8+SB, and control groups. The results of three separate experiments were presented as mean ± SD. *n* = 8 mice in each group. ^#^*p* < 0.05, compared to the CFA group; *p < 0.05, compared to the control group; ^$^*p* < 0.05, compared to the A8 group.

NFκB and PI3K, well-known key immune sensors for the innate immune response to produce robust inflammation [10], were examined in the control, CFA, A8, and A8+SB groups. As shown in Fig. 7A, NFκB p65 expression was significantly decreased after CFA induction (36.1 ± 7.7), but increased after A8 treatment (125.6 ± 11.0, *p* = 0.0210). However, this effect was reversed when A8 was co-administrated with SB (31.0 ± 8.9, *p* = 0.0314). The PI3K and AKT expression displayed a trend similar to that of NFκB p65 (all *p* < 0.05) (Fig. 7B, C). In addition, WB analysis also confirmed the changes in NFκB, PI3K, and AKT expression. The data also showed that the phosphorylation of NFκB and AKT was reduced after CFA treatment, and then, increased following A8 administration. However, their phosphorylation was clearly decreased when SB was co-treated with A8 (Fig. 7D), indicating that NFκB, PI3K, and AKT components were involved in the TRPV1-mediated CFA-induced inflammatory pain.

## Discussion

It is difficult to evaluate inflammatory pain levels due to knowledge gaps in terms of pathogenesis, as well as acute expansion and localization. Currently, CFA-induced inflammatory pain is assessed via behavior examinations, such as narcosis efficacy, the occurrence of abdominal contractions, or AWR [11], however, all these diagnostic approaches are considered inadequate. Inflammatory pain is owned by the category of chronic pain, which is a persistent pain state caused by peripheral tissue damage mediated by a variety of factors (including trauma, infection caused by bacteria or virus, and surgery, etc.,) and is one of the most common types of clinical pain [12]. Inflammatory pain diseases often occur simultaneously with other diseases (trauma, rheumatoid arthritis, osteoarthritis, etc.) and show complex paralgesia and hyperalgesia. CFA is often used in the induction of various chronic inflammatory pain models [13]. The present research revealed that CFA activation in mice resulted in acute inflammatory pain. Furthermore, α2-adrenoceptor antagonist A8 treatment repressed behavioral visceromotor reactions to pain and inhibited acute inflammation reactions. It was also found that CFA stimulation reduced TRPV1 expression whereas A8 administration augmented TRPV1 activity. The co-treatment of A8 and TRPV1 activator SB counteracted the effect of A8 solo treatment on pain behaviors and inflammation. The data suggested that the antinociceptive effect of A8 in the CFA-treated mouse model was exhibited in a TRPV1-dependent manner.

Inflammation pain assessment commonly relies on various behavioral reactions, including AWR, analgesic utilization, and abdominal contraction records, which indicate circumstances of inflammation pain [14]. Long-term neural plasticity occurs in not only the brain but also the spinal cord. Allodynia (repressed pain threshold, pain triggered via painless triggers) and hyperalgesia (extended pain receptive field, reinforced reactions to nociceptive triggers, and extended pain time) is frequently seen. It has been previously proved that CFA treatment leads to remarkable mechanical and thermal hyperalgesia [13,15,16]. The present research assessed pain behaviors with the help of MWT, AWR, and TWL values in inflammation pain murine models triggered via CFA supplement, and discovered that AWR scores were remarkably elevated, while the other two were remarkably repressed in the disease group in comparison with a control group, suggesting that inflammation pain model was successfully established.

TRPV1 receptors were discovered on peripheral and central endings previously [17], and acted as integrators of nociceptive triggers. Increasing evidence has demonstrated an essential contribution of TRPV1 in the modulation of pain and inflammatory reactions [18,19]. Previous study suggested that transient hyperalgesia could be triggered, at least in part, through ion channel 3 pathway (acid-sensitive) after TRPV1 was knocked out [20]. AM404, a dual agonist of TRPV1 and Cannabinoid Receptor type 1, has been proven to prohibit the NFAT and NFκB pathways and to reduce migration and invasion of neuroblastoma cells [21]. Feedback regulation of JNK1 stimulation via NFκB regulates TRPV1-triggered promotion of IL-8 and IL-6 generation via human corneal epithelial cells [22]. TRPV1 was upregulated in acute human inflammatory bowel diseases and experimental colitis. Matsushita and colleagues have revealed that DEX exposure to murine DRG neurons represses capsaicin current amplitude, indicating that DEX supplement directly represses TRPV1 stimulation [23]. Furthermore, DEX efficiently has been reported to reduce cumene hydroperoxide/ADP-ribose-triggered TRPM2 and TRPV1 densities in the neurons of mice with cerebral ischemia [24]. The present findings demonstrated TRPV1 downregulation as well as simultaneous repression of CGRP generation in a murine inflammatory pain model; however, α2-adrenoceptor antagonist A8 treatment remarkably upregulated TRPV1. The present results indicated that TRPV1 could serve as a downstream regulator of A8 activities in murine models. Nevertheless, how CFA repressed TRPV1 expression as well as stimulation needs more comprehensive exploration.

## Conclusion

Taken together, this study showed that A8 treatment inhibits CFA-triggered inflammatory pain and exerts a significant inhibitory effect on the production of pain-associated pro-inflammatory cytokines in a mouse model. Administration of A8 also increased the signal transduction of the TRPV1 channel. In addition, the present study suggests that the antinociceptive effect of A8 may be regulated by suppressing the inflammatory response via NFκB and PI3K activation.

## Authors’ contributions

Xiaomei Ling: Conceptualization, Data curation, Formal analysis, Investigation, Validation, Writing - original draft. Wei Wang: Conceptualization, Data curation, Formal analysis, Investigation, Validation, Writing - review & editing. All authors approved the final manuscript.

## Funding

This research did not receive any specific grant from funding agencies in the public, commercial, or not-for-profit sectors.

## Conflicts of interest

The authors declare no conflicts of interest.
